# Identification and validation of potential key long noncoding RNAs in sorafenib-resistant hepatocellular carcinoma cells

**DOI:** 10.7717/peerj.8624

**Published:** 2020-02-26

**Authors:** Manya Wu, Xiaoyun Shen, Yanping Tang, Caifu Zhou, Haixia Li, Xiaoling Luo

**Affiliations:** 1 Research Department, Guangxi Medical University Cancer Hospital, Nanning, China; 2 Guangxi Medical University, Nanning, China; 3 Department of Experimental Research, Sir Run Run Shaw Hospital, School of Medicine, Zhejiang University, Hangzhou, China

**Keywords:** Hepatocellular carcinoma, Global transcriptomic, Sorafenib-resistance, Long noncoding RNA

## Abstract

As the first-line treatment, sorafenib has been used for advanced hepatocellular carcinoma (HCC), but the chemoresistance commonly restricts to the clinical efficiency. In this study, we intend to investigate the genome-wide expression pattern of long noncoding RNAs (lncRNAs) in sorafenib-resistant HCC. Herein, we identified thousands of differentially expressed lncRNAs in sorafenib-resistant HCC cells by high-throughput sequencing compared to the parental. Besides, based on GO (Gene Ontology) term enrichment analysis, these differentially expressed lncRNAs are mainly related to binding and catalytic activity and biological regulation of metabolic processes in both the sorafenib-resistant Huh7 cells (Huh7-S) and sorafenib-resistant HepG2 cells (HepG2-S) compared to the parental cells. Moreover, when analyzed by KEGG (Kyoto Encyclopedia of Genes and Genomes) pathway, the differentially expressed genes were significantly related to the tight junction. Among them, the expression of TCONS_00284048 and TCONS_00006019 was consistently up-regulated in sorafenib-resistant HCC cell lines, whereas when either was knocked down, the sensitivity of Huh7-S and HepG2-S cells to sorafenib was increased. Taken together, our data demonstrate that the lncRNA expression profile is significantly altered in sorafenib-resistant HCC cells as well as differentially expressed lncRNAs may play crucial functions on HCC sorafenib resistance and HCC progression.

## Introduction

With more than 780,000 deaths annually, HCC is the fourth main cause of cancer death globally (Bray et al., 2018). Though surgical resection is still the preferred method for the treatment of liver cancer, most of patients are at the advanced stage of disease at the time of diagnosis and thus are unsuitable for resection. Sorafenib, as an oral multi-kinase inhibitor, is the unique molecular targeted drug used to treat with advanced HCC patients, for whom the resection is not an option (Lo et al., 2015). Although sorafenib-treated patients have been demonstrated with an increased median survival time (Cheng et al., 2009; Llovet et al., 2008), the high level of resistant rate has limited the efficiency of sorafenib therapy significantly.

Long noncoding RNAs (lncRNAs), a member of non-coding RNA transcripts, are more than 200 nucleotides in size. They have been classified into not only sense lncRNAs, antisense lncRNAs and intergenic lncRNAs, but intronic lncRNAs and bidirectional lncRNAs (Derrien et al., 2012; Huo et al., 2017). In these years, lncRNAs have been as an important role in various human cancers development and progression (Chen et al., 2017; Hanahan & Weinberg, 2000). For instance, by sponging miR-140-5p, lncRNA PVT1 could promote the cervical cancer proliferation and metastasis through increasing the Smad3 expression (Chang et al., 2019). In addition, upregulating insulin growth factor-binding protein 2, lncRNA HOTAIR in renal cell carcinoma could induce cell migration (Katayama et al., 2017) as well as lncRNA CCAT5 promotes tumor growth, invasion, and metastasis through upregulating STAT3 in colorectal cancer (Wang, Yan & Tian, 2019). Moreover, in HCC, dysregulation of lncRNAs, including HULC (Wang et al., 2017), UFC1 (Cao et al., 2015), ZNRD1 (Wen et al., 2015), MEG3 (Zhang et al., 2019), PTENP1 (Qian et al., 2017) have been demonstrated to regulate cell growth and proliferation, H19 (Li et al., 2019a), MALAT1 (Huang et al., 2018), ATB (Yuan et al., 2014), HEIH (Yang et al., 2011) have reported participating in invasion and metastasis, SPRY4-IT1 (Zhou, Zhang & Yu, 2017), SNHG20 (Liu et al., 2017), UCA1 (Xiao et al., 2017) have found to involve in epithelial to mesenchymal transition (EMT). Furthermore, a group of lncRNAs have been investigated to participate in cancer chemoresistance. For instance, lncRNA HOTTIP can promote gemcitabine resistance in pancreatic cancer (Li et al., 2015), lncRNA CCAT1 can promote docetaxel resistance in lung adenocarcinoma by sponging miR-let-7 (Chen et al., 2016), linc00152 can contribute to oxaliplatin resistance through sponging miR-193a-3p in colon cancer (Yue et al., 2016).

Though the role of lncRNAs in the onset of the disease has received attention, research into the relationship between lncRNAs and chemo-resistance, particularly in sorafenib-resistant HCC, is rare. Herein, we analyzed the differential expression profiles of lncRNAs in sorafenib-resistant HCC cells and investigated the relationship between the lncRNA expression and sorafenib-resistance to provide a preliminary and theoretical basis for the identification of biomarkers for the diagnosis prognosis, chemosensitivity, and treatment of HCC.

## Materials & Methods

### Cell culture

The human HCC cell lines Huh-7 and HepG2 were obtained from the Shanghai Institute of Biochemistry and Cell Biology of Chinese Academy of Science. Supplemented with 10% fetal bovine serum, Huh-7 cells cultures were maintained in RPMI-1640 while HepG2 cells were cultured in Eagle’s Minimum Essential Medium. All of cells were kept in 37 °C, humidified incubator containing 5% CO_2_ for culture.

### Generation of drug-resistant cells

Cells were treated with 1.5 µM sorafenib (Selleck Chemicals, Houston, TX, USA) after plating into a 6 cm cell culture dish (1 × 10^5^ cells per dish) for 24 h. The viable cells still attached to the dish respectively were treated with various concentrations (1.5, 3.0, 4.5, 6.0, 7.5 and 9.0 µM) and maintained for 15-21 days. To the end of the fifth month, the cells were becoming stable resistant to sorafenib and re-named Huh-7-S and HepG2-S cells. All experiments were performed in triplicate.

### Cell viability assay

Cells were seeded in 96-well plate at 1 × 10^4^ cells per well and then treated with sorafenib at concentrations ranging from 0 to 27 µM. Viable cells were quantified using the Cell Counting Kit-8 (Dojindo Chemical, Kumamoto, Japan) after 72 h. Absorbance was measured at 450 nm using a microplate reader (Corning, USA) with a reference wavelength of 600 nm. The IC50 values of sorafenib pretreatment on cell viability were examined by the CCK8 assay.

### RNA isolation and qualification

TRIzolTM reagent (Invitrogen, USA)/RNeasy Mini Kit (Qiagen) was used to isolate total RNA. RNA concentration was measured by Qubit® 2.0 Fluorometer (Life Technologies, CA, USA) with Qubit® RNA Assay Kit. RNA intergrity was performed with the RNA Nano 6000 Assay Kit after testing RNA purity by NanoPhotometer® spectrophotometer from Agilent Technologies (CA, USA). Deep sequencing was conducted using only samples (1 µg total RNA) with an RNA integrity number (RIN) of ≥ 7. NEBNext® Ultra™ Directional RNA Library Prep Kit from Illumina® was used in order to prepare for the Next-generation sequencing library.

### Strand-specific RNA-seq library preparation & sequencing

In order to prepare a strand-specific RNA-sequencing library with NEBNext® UltraTM Directional RNA Library Prep Kit from Illumina® (NEB, USA), ribosomal RNA was depleted by using Ribo-Zero™ rRNA Removal Kit before fragmented and reverse transcribed. The first-strand cDNA was briefly synthetized using ProtoScript II Reverse Transcriptase with random primers and Actinomycin D. Second Strand Synthesis Enzyme Mix (include dACG-TP/dUTP) was acquired to synthetize the second-strand cDNA. The purified double-stranded cDNA using AxyPrep Mag PCR Clean-up (Axygen) was then treated with End Prep Enzyme Mix for the reparation of both ends and add a dA-tailing, before adding T-A ligation adaptors to both ends. Adaptor-ligated DNA then went through size selection followed by recovering the fragments of ∼360 bp. Then, Uracil-Specific Excision Reagent (USER) enzyme from New England Biolabs was required for the dUTP-marked second strand digestion. Amplifying each sample by PCR was done at 11 cycles with P5 and P7 primers. Sequences of adaptors were as, P7 adapter, 5′-AGATCGGAAGAGCACACGTCTGAACTCCAGTCAC-3′; P5 adapter, 5′-AGATCGGAAGAGCGTCGTGTAGGGAAAGAGTGT-3′.

### Read alignment and transcript assembly

The original image data of the sequencing result was subjected to image base recognition (Base Calling) using the software Bcl2fastq (v2.17.1.14), with preliminary quality analysis. The original sequencing data (Pass Filter Data) was obtained, and the result was stored in the FASTQ file format. The quality of the sequencing data was analyzed using the software FastQC (v0.10.1). Subsequent information analysis was performed after obtaining clean data without the linker and low-quality results by using the data quality statistical software Cutadapt (v1.3) for second-generation sequencing. The clean data was compared to the reference genome sequence using HISAT2 (v2.0.1) alignment software, and the results were compared. Finally, all the alignments for transcript assembly were passed to the software Stringtie (v1.0.4).

### Bioinformatics analysis

Differential expression analysis was carried out by using the DESeq (V1.18.0), DESeq2 (V1.6.3) and EdgeR (V3.4.6) Bioconductor package. The Benjamini and Hochberg approach was used to control the false discovery rate. The *p*-value was set to *P* < 0.05 to detect differentially expressed genes. Gene Ontology (GO) terms were identified with a list of enriched genes by using GO-TermFinder (V0.86) as well as KEGG pathway analysis was used to harvest pathway clusters in differentially expressed gene profiling.

### Quantitative RT-PCR

Real-time PCR was carried out by using SYBR Premix Ex Taq from TaKaRa on a LightCycler480 instrument (Roche Diagnostics) and data were analyzed by using relative quantification (2^−ΔΔ*Ct*^). The used primer sequences were as follows: TCONS_00284048 forward, 5′-AATCGTTCCACAACGCGGAG-3′; reverse, 5′-GAGGGCTTGGGTTAGAGCTAC-3′; TCONS_00006019 forward, 5′-TGATGCACTCAGCAGTTCCC-3′; reverse, 5′-GCCAGAGGATCCAGGTGTACAT-3′. GAPDH was an internal normalized control. All samples were performed by qPCR in triplicate.

### Cell transfection

For the gene knockdown experiments, siRNAs were all from Gene Pharma Company (Shanghai, China) and the sequences contained were as follows: si-TCONS_00284048, sense, 5′-GGUCUUGCUGCUUCCUUUA-3′; antisense, 5′-UAAAGGAAGCAGCAAGACC-3′; si-TCONS_00006019: sense, 5′-GAGGAAGGAACUAAUGUAA-3′; antisense, 5′-UUACAUUAGUUCCUUCCUC-3′; Transfections were carried out by using lipofectamine 2000 reagent from Invitrogen according to manufacturer’s instructions.

### Statistical analysis

The statistical analysis involved using GraphPad Prism 6.0. The comparison of differences between the two groups was performed by using Independent Student *t* test. LncRNAs with a fold change of more than 2 and *P* values < 0.05 were considered statistically significant. All experiments contained were performed three times.

## Results

### Establishment of sorafenib-resistant HCC cells

**Figure 1:**
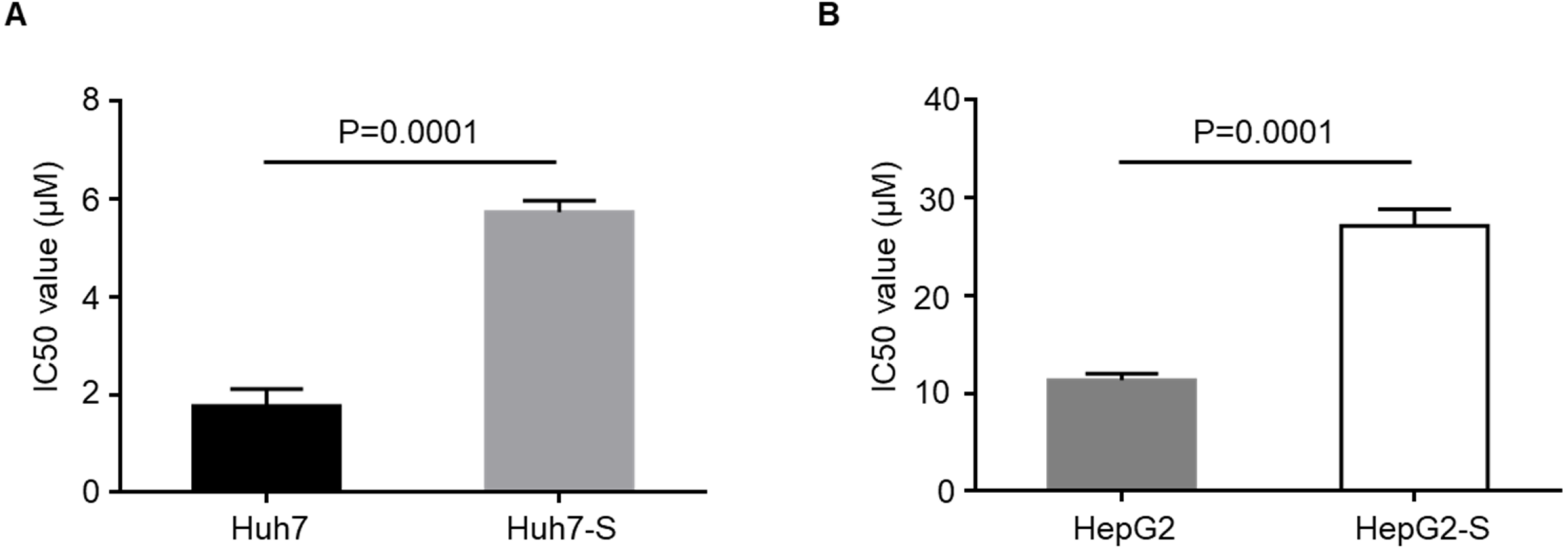
Resistance of HCC cell lines to sorafenib. (A) The effects of Huh7 cells and sorafenib-resistant Huh7 cells to sorafenib were assessed by CCK8 assay. (B) The effects of HepG2 cells and sorafenib-resistant HepG2 cells to sorafenib were assessed by CCK8 assay. Data are shown as mean ± SEM of three independent experiments.

IC50 values were to determine the efficacy of sorafenib resistance in Huh7 and HepG2 cells. The values IC50 were significantly higher in sorafenib resistant cell lines, with Huh7-S cells (IC50 = 5.73  ± 0.23 µM) showing a 3.24-fold increased resistance to sorafenib compared to parental Huh7 cells (IC50 = 1.77  ± 0.35 µM; *p* = 0.0001) (Fig. 1A) and HepG2-S cells (IC50 = 27.09 ± 1.73 µM) showing a 2.38-fold increased resistance compared to parental HepG2 cells (IC50 = 11.36 ± 0.66 µM; *p* = 0.0001) (Fig. 1B). Thus, sorafenib-resistant HCC cells were successfully established.

### Screening of differentially expressed lncRNA

**Figure 2:**
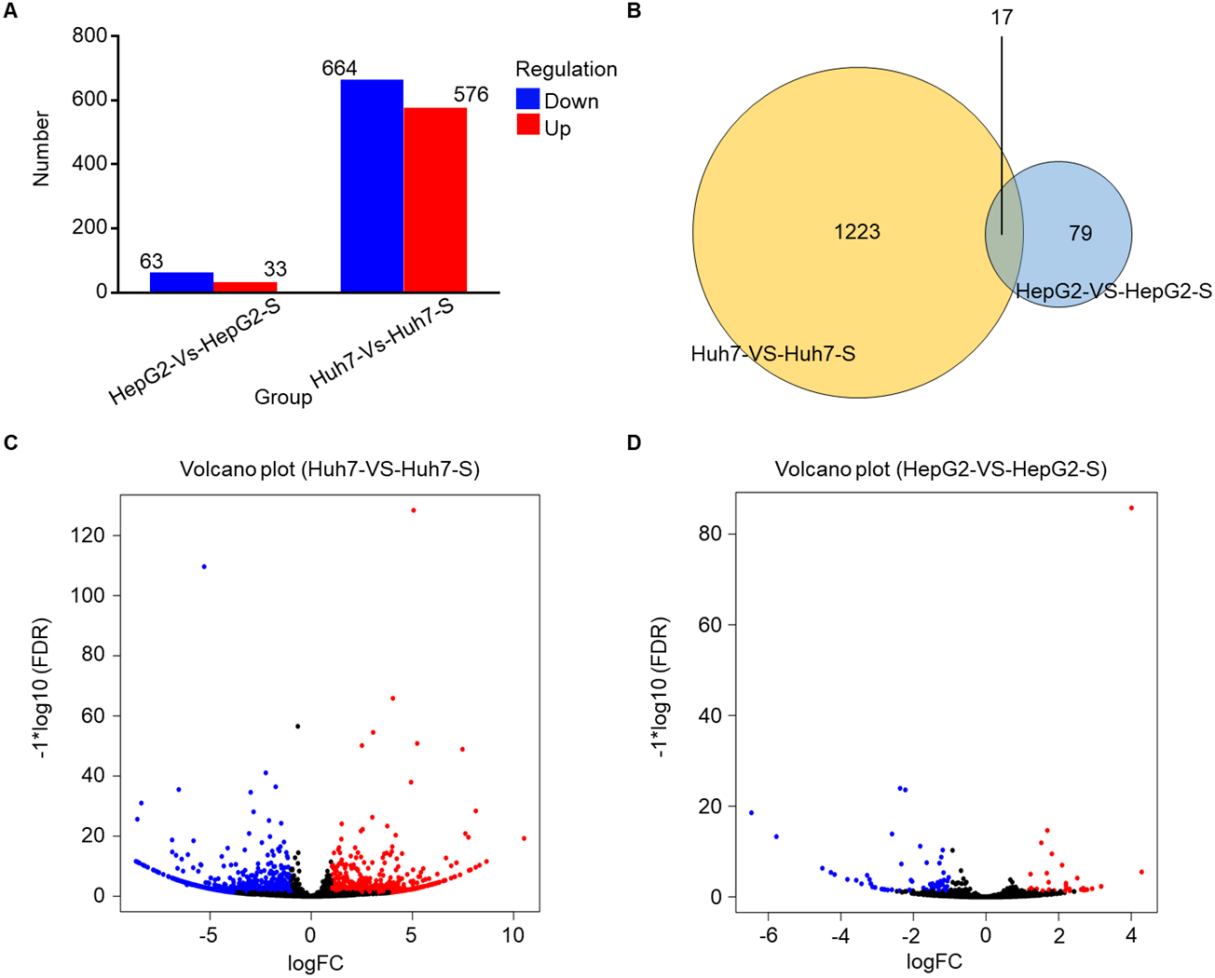
Screening of differentially expressed lncRNAs. (A) The number of differentially expressed lncRNAs in sorafenib-resistant Huh7 and HepG2 cells compared to the parental cells (blue, down-regulated; red, up-regulated). (B) Venn diagrams of differentially expressed lncRNAs in sorafenib-resistant Huh7 and HepG2 cells compared to the parental cells. (C) Volcano plots of differentially expressed lncRNAs between Huh7 and Huh7-S cells. (D) Volcano plots of differentially expressed lncRNAs between HepG2 and HepG2-S cells.

**Figure 3:**
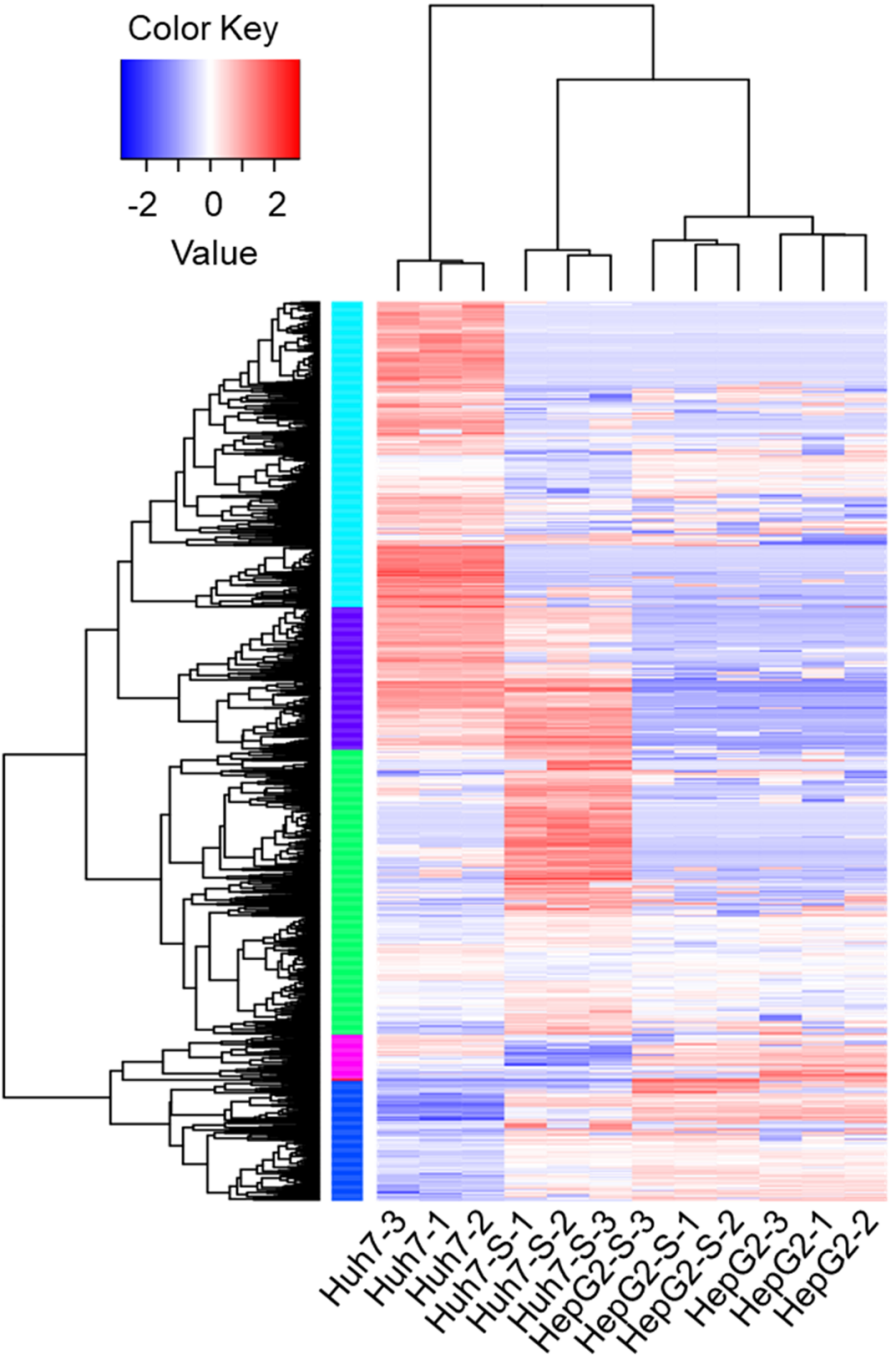
The expression profiles of lncRNAs. Heat map was performed to demonstrate the hierarchical clustering of lncRNA expression in sorafenib-resistant Huh7 and HepG2 cells compared to the parental cells. The color depth indicated the elevated (Red) and decreased (Blue) expression level across all samples.

Using an Illumina HiSeq instrument, the lncRNA expression profile of sorafenib-resistant HCC cells was assessed. As shown in Fig. 2A, in Huh7-S cells, there were 1,240 differentially expressed lncRNAs compared to parental Huh7 cells, with 576 up-regulated and 664 down-regulated lncRNAs. Interestingly, there were only 96 differentially expressed lncRNAs in HepG2-S cells compared to parental HepG2 cells, with 33 up-regulated and 63 down-regulated. Visualization of the differentially expressed lncRNAs using Venn diagrams revealed 17 common lncRNAs between the Huh7 and HepG2 comparisons (Fig. 2B). The number of significantly altered lncRNAs (log fold change <−1 or >1 and *p* < 0.05) was visualized using a volcano plot (Figs. 2C and 2D). Hierarchical clustering analysis shows a distinguishable lncRNA expression profile among samples (Fig. 3).

### Prediction and functional classification of target genes

**Figure 4:**
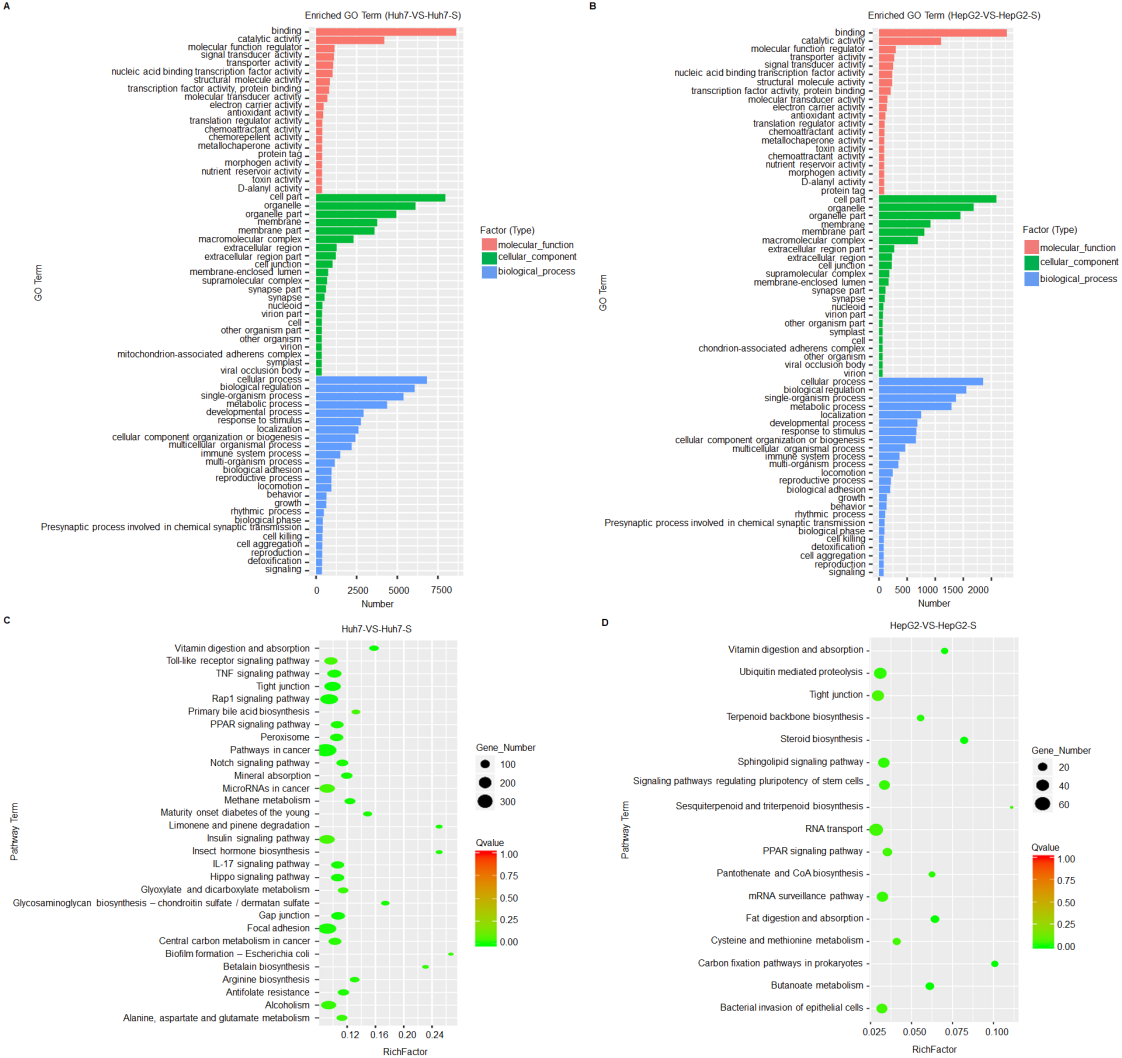
Prediction and functional classification of target genes. (A) GO enrichment analysis of significantly differential expressed lncRNAs between Huh7 and Huh7-S cells. (B) GO enrichment analysis of significantly differential expressed lncRNAs between HepG2 and HepG2-S cells. (C) KEGG pathway analysis of significantly differential expressed lncRNAs between Huh7 and Huh7-S cells. (D) KEGG pathway analysis of significantly differential expressed lncRNAs between HepG2 and HepG2-S cells.

Based on GO term enrichment analysis, the differentially expressed lncRNAs are mainly related to binding and catalytic activity and biological regulation of metabolic processes in the sorafenib-resistant cell lines, compared to parental cell lines (Figs. 4A and 4B). KEGG molecular functions identified a large proportion of lncRNAs involved in tight junction common to both cell line comparisons (Figs. 4C and 4D).

### Validation of differentially expressed lncRNAs

**Figure 5:**
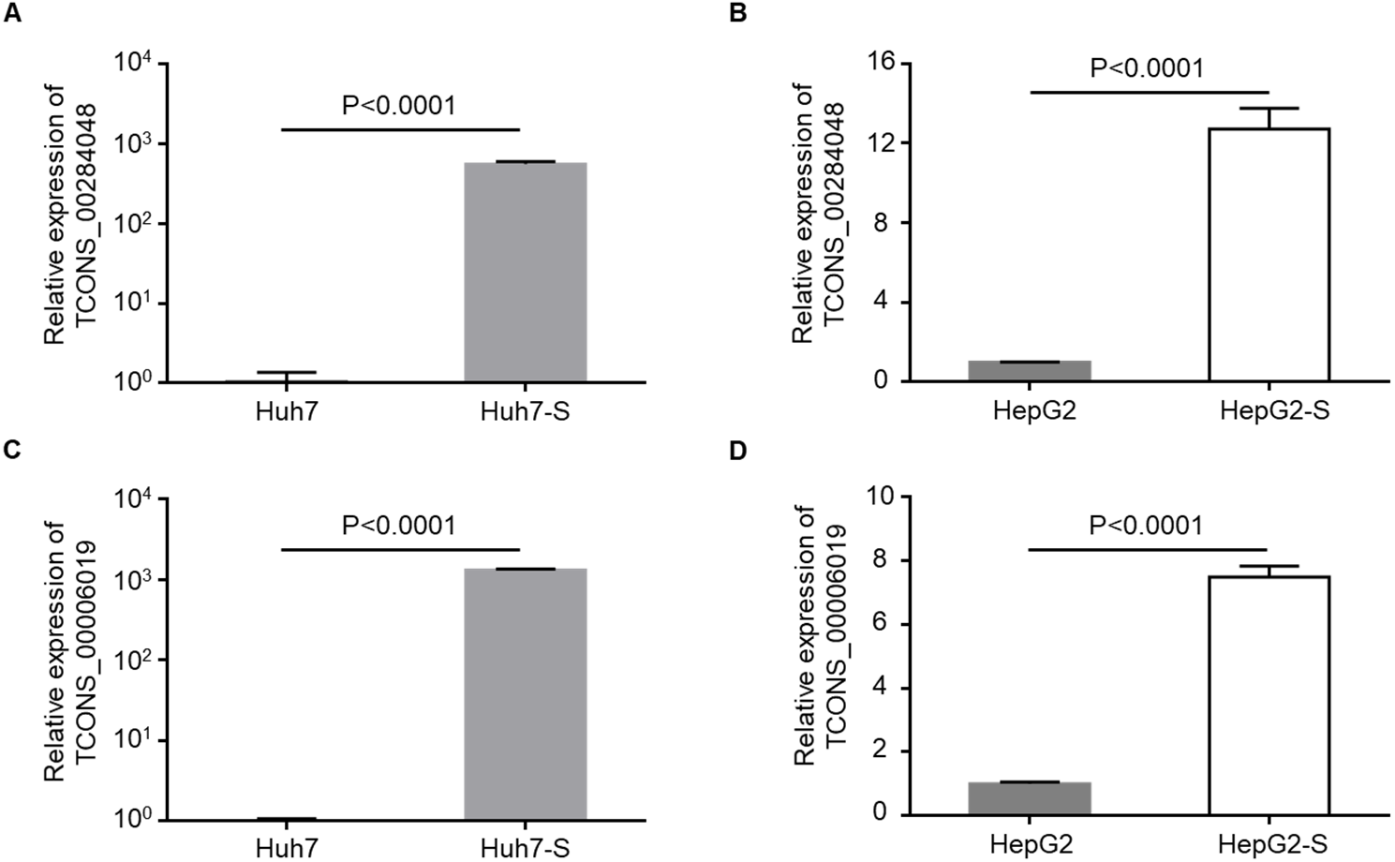
Validation for lncRNAs expression. (A) The expression level of TCONS_00284048 was validated using RT-qPCR in sorafenib-resistant Huh7 cells. (B) The expression level of TCONS_00284048 was validated in sorafenib-resistant HepG2 cells. (C) The expression level of TCONS_00006019 was validated in sorafenib-resistant Huh7 cells. (D) The expression level of TCONS_00006019 was validated in sorafenib-resistant HepG2 cells. *p* < 0.0001 compared to parental controls. Data are shown as mean ± SEM of three independent experiments.

Two lncRNAs, TCONS_00284048 and TCONS_00006019, were up-regulated in sorafenib-resistant cell lines compared to parental control cell lines, and were selected for use in further validation experiments. qPCR data confirmed that the expression levels of TCONS_00284048 (Figs. 5A and 5B) and TCONS_00006019 (Figs. 5C and 5D) were significantly increased in both Huh7-S (*p* < 0.0001) and HepG2-S (*p* < 0.0001) cell lines compared to parental controls, indicating that our deep sequencing data are reliable.

### Function of differentially expressed lncRNAs

**Figure 6:**
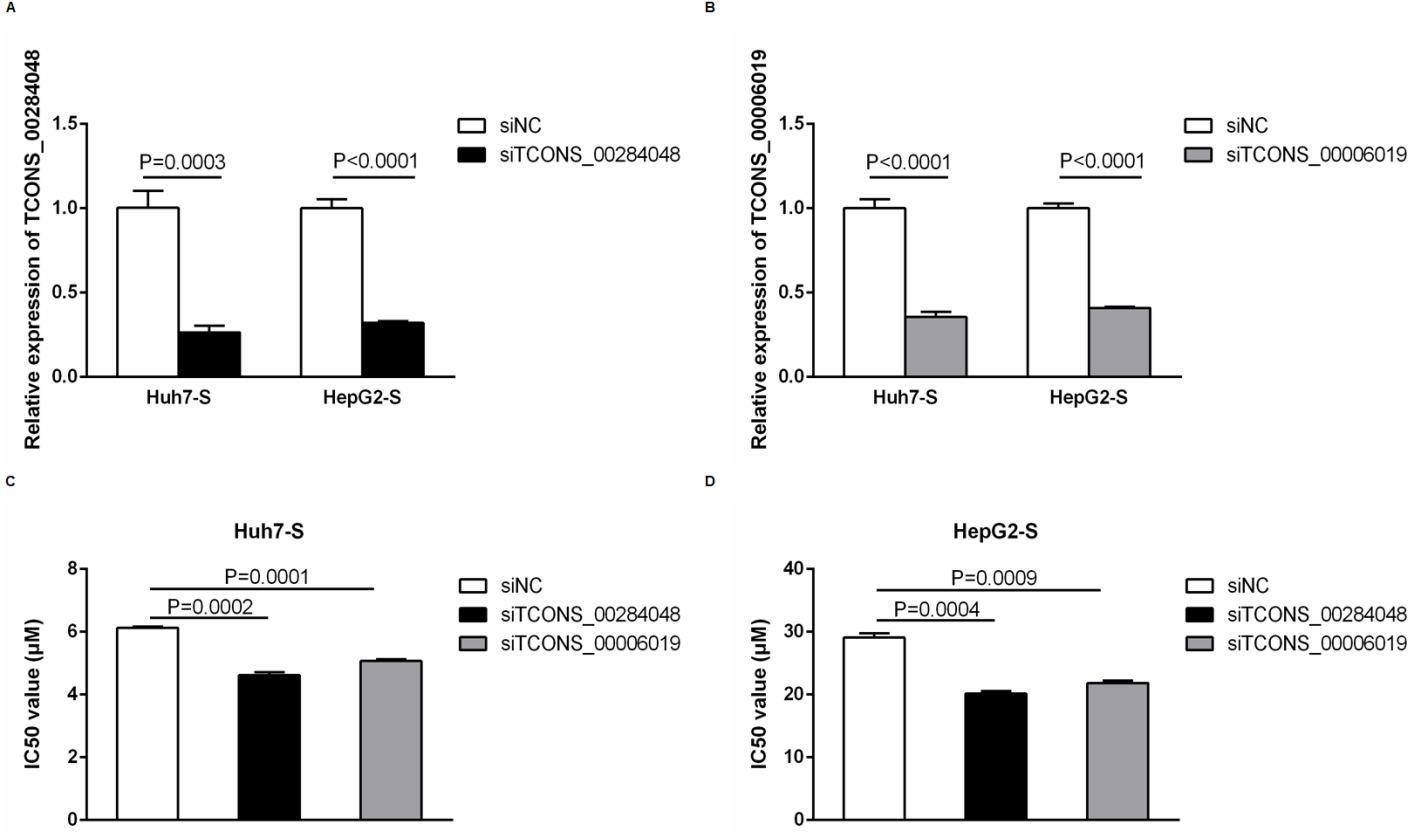
Function of differentially expressed lncRNAs. (A) The expression levels of TCONS_00284048 as silenced using siRNA in both sorafenib-resistant Huh7 and HepG2 cells. (*p* = 0.0003 and *p* < 0.0001 respectively). (B) The expression levels of TCONS_00006019 as silenced using siRNA in both sorafenib-resistant Huh7 and HepG2 cells (*p* < 0.0001). (C) IC50 values were significantly lower in sorafenib resistant Huh7 cells after knockdown TCONS_00284048 or TCONS_00006019, compared to the siNC Huh7-S cells (*p* = 0.0002 and *p* = 0.0001 respectively). (D) IC50 values were significantly lower in sorafenib resistant HepG2 cells after knockdown TCONS_00284048 or TCONS_00006019, compared to the siNC HepG2-S cells (*p* = 0.0004 and *p* = 0.0009 respectively). Data are shown as mean ± SEM of three independent experiments.

In order to further explore the involvement of lncRNAs in sorafenib-resistant HCC, we used siRNA to knockdown TCONS_00284048 and TCONS_00006019 respectively in both Huh7-S and HepG2-S cells. The expression of TCONS_00284048 (Fig. 6A) and TCONS_00006019 (Fig. 6B) was effectively silenced using siRNA (*p* = 0.0003 and *p* < 0.0001 respectively). It was observed that the expression of TCONS_00284048 or TCONS_00006019 was significantly downregulated, 48 h after transfection with siTCONS_00284048 or siTCONS_00006019 by Lipo2000 in mRNA levels as compared with the siNC control. In order to determine the efficacy of sorafenib resistance in Huh7 and HepG2 cells after knockdown TCONS_00284048 or TCONS_00006019, IC50 values of sorafenib-resistant HCC cell lines were determined by CCK-8 assay. Huh7-S transfected with siTCONS_00284048 (IC50 = 4.62 ± 0.10 µM) and transfected with siTCONS_00006019 (IC50 = 5.07 ± 0.06 µM) showing a 0.75-fold and 0.83-fold respectively decreased resistance to sorafenib compared to that with transfected siNC Huh7-S cells (IC50 = 6.13 ± 0.04 µM; *p* = 0.0002 and *P* = 0.0001 respectively) (Fig. 6C). HepG2-S cells transfected with siTCONS_00284048 (IC50 = 20.15 ± 0.40 µM) and transfected with siTCONS_00006019 (IC50 = 21.85 ± 0.38 µM) showing a 0.69-fold and 0.75-fold respectively decreased resistance to sorafenib compared to that with transfected siNC HepG2-S cells (IC50 = 29.07 ± 0.72 µM; *p* = 0.0004 and *p* = 0.0009 respectively) (Fig. 6D). Our data indicated that both TCONS_00284048 and TCONS_00006019 significantly enhanced sorafenib resistance in HCC cells.

## Discussion

Sorafenib, the most widely used drug for patients with non-resectable tumors, is the only FDA-permitted systemic therapy for HCC in the clinical first line (Ray & Sanoff, 2017). In addition to targeting multiple growth factor receptors, such as EGFR and VEGFR, sorafenib has also shown to inhibit different isoforms of Raf kinase potently (Liu et al., 2006). Although the advanced HCC patients who treated with sorafenib could have a survival advantage of 2–3 months, the development of its resistance greatly minimizes its therapeutic benefits (Cheng et al., 2009; Llovet et al., 2008). It is generally believed that affecting DNA damage repair, EMT, or regulation of signaling pathway, such EGFR and its downstream signaling molecules (Zhang et al., 2009), PI3K/Akt (Morgensztern & McLeod, 2005) and JAK-STAT (Chen et al., 2012) pathways may contribute to sorafenib resistance. Therefore, it is imperatively required to further explore the mechanisms of sorafenib resistance and seek for more novel molecular biomarkers then to increase the rate of early diagnosis of HCC.

Recently, lots of lncRNAs have been involved in various human cancers progression and participated in cancer drug resistance, and the potential mechanisms of action in the majority of them are being revealed gradually. For example, lncRNA HOTAIR has been reported to promote the DNA-damaging drug cisplatin resistance in part by regulating p21^WAF1/CIP1^ pathways in lung cancer cell lines (Liu et al., 2013). Besides, lncRNA UCA1 leads to cisplatin resistance through the upregulation of the Wnt pathway (Fan et al., 2014). Chen et al. (2019) found lncRNA HIF1A-AS2 might target in hypoxia-mediated therapeutic resistance via HMGA1/p53 pathway in bladder cancer. Further, lncRNAs also have been identified as regulators of sorafenib resistance. Activating the Akt pathway, lncRNA SNHG1 induces sorafenib resistance in HCC cells (Li et al., 2019b). Sponging miR-335, lncRNA NEAT1 suppresses the c-MET and AKT pathway thereby increasing HCC cells’ sensitivity to sorafenib (Chen & Xia, 2019).

In our study, differentially expressed lncRNAs have been explored in sorafenib-resistant HCC cell lines through high-throughput sequencing and validated two of them, TCONS_00284048 and TCONS_00006019 expression were consistently up-regulated in resistant HCC cells, whereas both of them could enhance the resistance of Huh7-S and HepG2-S cells to sorafenib. TCONS_00284048, known as HNF4A-AS1, has been reported to have high expression not only in duodenum and ileum, but Caco-2 cell line. In addition to decreased expression in neutrophils, TCONS_00284048 has no expression in other humanized blood-derived cells and intestinal tissues (Haberman et al., 2018). TCONS_00006019 is the novel lncRNA, also named AL109659.2, affiliated with the antisense to SLC5A9. SLC5A9 encodes sodium-dependent glucose transporter 4 (SGT4), which is expressed in several tissues, such as kidney, liver, lung, brain, small intestines, heart (Wright, 2013; Wright & Turk, 2004), and plays a significant role in intestinal absorption and renal reabsorption of mannose (Tazawa et al., 2005). Since there are no studies about these two lncRNA in human cancer even cancer drug resistance, our findings might potentially provide two novel biomarkers in the diagnosis prognosis, chemosensitivity, and treatment of HCC.

## Conclusions

Our data demonstrated that lncRNA expression profile was significantly altered in sorafenib-resistant HCC cells. Furthermore, we found lncRNA HNF4A-AS1 and lncRNA AL109659.2 are upregulated in sorafenib-resistant HCC cells, which might be served as a chemotherapy indicator for advanced HCC patients. Additionally, we identified these differentially expressed lncRNAs are mainly related to binding and catalytic activity and biological regulation of metabolic processes using bioinformatics analysis. Therefore, these findings provide evidence that the lncRNA profile of HCC becomes altered during sorafenib resistance and may be of use as potential biomarkers of HCC patients who are developing sorafenib resistance. Thus, these differentially expressed lncRNAs involved in HCC sorafenib resistance should be required to explore the biological function and molecular mechanism in further studies. It will be of great importance in liver cancer research to investigate the occurrence and development of HCC, including the identification of new biomarkers for the diagnosis prognosis, chemosensitivity, and treatment.

##  Supplemental Information

10.7717/peerj.8624/supp-1 Supplemental Information 1 Raw data of validation of differentially expressed lncRNAs Real-time PCR was performed using SYBR Premix Ex Taq (TaKaRa) on a LightCycler480 instrument (Roche Diagnostics) according to the manufacturer’s protocol. GAPDH was used as an internal normalized reference, and fold changes were calculated by relative quantification (2^−ΔΔ*Ct*^). The expression of seven lncRNAs, TCONS_00215594, TCONS_00052270, TCONS_00284048, TCONS_00398166, TCONS_00389094 (Sheet1), TCONS_00006019 (Sheet2) and TCONS_00046965 (Sheet3) were run in both Huh7-S and HepG2-S cell lines compared to parental control cell lines. TCONS_00284048 and TCONS_00006019 were selected for use in further validation experiments.

10.7717/peerj.8624/supp-2 Supplemental Information 2 Raw data of the knockdown efficiency of TCONS_00284048 in HepG2-S cell For the TCONS_00284048 knockdown experiments, siRNA was designed and synthesized by Gene Pharma Company (Shanghai, China) and the sequences were as follows: TCONS_00284048siRNA1:GGTCTTGCTGCTTCCTTTA; TCONS_00284048siRNA2: GATATGAATCGTTCCACAA; TCONS_00284048siRNA3:ACACCGGTCATATTATTGA. Transfections were carried out using lipofectamine 2000 reagent (Invitrogen) according to manufacturer’s protocol.The expression of TCONS_00284048 in sorafenib-resistant HepG2 cell lines (HepG2-S) were examined after transfected with siRNA knockdown system compared with the negative control (siRNA NC). Real-time PCR was performed using SYBR Premix Ex Taq (TaKaRa) on a LightCycler480 instrument (Roche Diagnostics) according to the manufacturer’s protocol. GAPDH was used as an internal normalized reference, and fold changes were calculated by relative quantification (2^−ΔΔ*Ct*^). TCONS_00284048siRNA1 was selected for use in further validation experiments.

10.7717/peerj.8624/supp-3 Supplemental Information 3 Raw data of knockdown efficiency of TCONS_00284048 in Huh7-S cell For the TCONS_00284048 knockdown experiments, siRNA was designed and synthesized by Gene Pharma Company (Shanghai, China) and the sequences were as follows: TCONS_00284048siRNA1:GGTCTTGCTGCTTCCTTTA; TCONS_00284048siRNA2: GATATGAATCGTTCCACAA; TCONS_00284048siRNA3:ACACCGGTCATATTATTGA. Transfections were carried out using lipofectamine 2000 reagent (Invitrogen) according to manufacturer’s protocol.The expression of TCONS_00284048 in sorafenib-resistant Huh7 cell lines (Huh7-S) were examined after transfected with siRNA knockdown system compared with the negative control (siRNA NC). Real-time PCR was performed using SYBR Premix Ex Taq (TaKaRa) on a LightCycler480 instrument (Roche Diagnostics) according to the manufacturer’s protocol. GAPDH was used as an internal normalized reference, and fold changes were calculated by relative quantification (2^−ΔΔ*Ct*^). TCONS_00284048siRNA1 was selected for use in further validation experiments.

10.7717/peerj.8624/supp-4 Supplemental Information 4 Raw data of knockdown efficiency of TCONS_00006019 in HepG2-S cell For the TCONS_00006019 knockdown experiments, siRNA was designed and synthesized by Gene Pharma Company (Shanghai, China) and the sequences were as follows: TCONS_00006019siRNA1:GGCACTGCATTCATAAATA; TCONS_00006019siRNA2: GAGGAAGGAACTAATGTAA; TCONS_00006019siRNA3:CCAAGTCTCAGAACTAGTA. Transfections were carried out using lipofectamine 2000 reagent (Invitrogen) according to manufacturer’s protocol.The expression of TCONS_00006019 in sorafenib-resistant HepG2 cell lines (HepG2-S) were examined after transfected with siRNA knockdown system compared with the negative control (siRNA NC). Real-time PCR was performed using SYBR Premix Ex Taq (TaKaRa) on a LightCycler480 instrument (Roche Diagnostics) according to the manufacturer’s protocol. GAPDH was used as an internal normalized reference, and fold changes were calculated by relative quantification (2^−ΔΔ*Ct*^). TCONS_00006019siRNA2 was selected for use in further validation experiments.

10.7717/peerj.8624/supp-5 Supplemental Information 5 Raw data of knockdown efficiency of TCONS_00006019 in Huh7-S cell For the TCONS_00006019 knockdown experiments, siRNA was designed and synthesized by Gene Pharma Company (Shanghai, China) and the sequences were as follows: TCONS_00006019siRNA1:GGCACTGCATTCATAAATA; TCONS_00006019siRNA2: GAGGAAGGAACTAATGTAA; TCONS_00006019siRNA3:CCAAGTCTCAGAACTAGTA. Transfections were carried out using lipofectamine 2000 reagent (Invitrogen) according to manufacturer’s protocol.The expression of TCONS_00006019 in sorafenib-resistant Huh7 cell lines (Huh7-S) were examined after transfected with siRNA knockdown system compared with the negative control (siRNA NC). Real-time PCR was performed using SYBR Premix Ex Taq (TaKaRa) on a LightCycler480 instrument (Roche Diagnostics) according to the manufacturer’s protocol. GAPDH was used as an internal normalized reference, and fold changes were calculated by relative quantification (2^−ΔΔ*Ct*^). TCONS_00006019siRNA2 was selected for use in further validation experiments.

## Abbreviation

HCC: hepatocellular carcinoma
lncRNA: long noncoding RNA
GO: Gene Ontology
KEGG: Kyoto Encyclopedia of Genes and Genomes
EMT: epithelial to mesenchymal transition
FBS: fetal bovine serum
CCK8: Cell Counting Kit-8
EGFR: epidermal growth factor receptor
VEGFR: vascular endothelial growth factor receptor
FDA: Food and Drug Administration
SGT4: sodium-dependent glucose transporter 4

## Additional Information and Declarations

### Data Availability

The following information was supplied regarding data availability: Data is available at GEO using accession number GSE140202.
